# High-impact exercise in adulthood and vertebral dimensions in midlife - the Northern Finland Birth Cohort 1966 study

**DOI:** 10.1186/s12891-017-1794-8

**Published:** 2017-11-06

**Authors:** Petteri Oura, Markus Paananen, Jaakko Niinimäki, Tuija Tammelin, Juha Auvinen, Raija Korpelainen, Jaro Karppinen, Juho-Antti Junno

**Affiliations:** 1 Medical Research Center Oulu, Oulu University Hospital and University of Oulu, P.O. Box 5000, 90014 Oulu, Finland; 2 Center for Life Course Health Research, Faculty of Medicine, University of Oulu, P.O. Box 5000, 90014 Oulu, Finland; 3 Research Unit of Medical Imaging, Physics and Technology, Faculty of Medicine, University of Oulu, P.O. Box 5000, 90014 Oulu, Finland; 4 LIKES-Research Center for Sport and Health Sciences, Rautpohjankatu 8, 40700 Jyväskylä, Finland; 5 Department of Sports and Exercise Medicine, Oulu Deaconess Institute, Albertinkatu 18A, 90100 Oulu, Finland; 6 Finnish Institute of Occupational Health, Kastelli Research Center, Aapistie 1, 90220 Oulu, Finland; 7 Cancer and Translational Medicine Research Unit, Faculty of Medicine, University of Oulu, P.O. Box 5000, 90014 Oulu, Finland; 8 Department of Archaeology, Faculty of Humanities, University of Oulu, P.O. Box 8000, 90014 Oulu, Finland

**Keywords:** Osteoporosis, Lumbar spine, Vertebral size, Sports participation, Magnetic resonance imaging, Cohort study

## Abstract

**Background:**

Vertebral size and especially cross-sectional area (CSA) are independently associated with vertebral fracture risk. Previous studies have suggested that physical activity and especially high-impact exercise may affect vertebral strength. We aimed to investigate the association between high-impact exercise at 31 and 46 years of age and vertebral dimensions in midlife.

**Methods:**

We used a subsample of 1023 individuals from the Northern Finland Birth Cohort 1966 study with records of self-reported sports participation from 31 and 46 years and MRI-derived data on vertebral dimensions from 46 years. Based on the sports participation data, we constructed three impact categories (high, mixed, low) that represented longitudinal high-impact exercise activity in adulthood. We used linear regression and generalized estimating equation (GEE) models to analyse the association between high-impact exercise and vertebral CSA, with adjustments for vertebral height and body mass index.

**Results:**

Participation in high-impact sports was associated with large vertebral CSA among women but not men. The women in the 'mixed' group had 36.8 (95% confidence interval 11.2–62.5) mm^2^ larger CSA and the women in the 'high' group 43.2 (15.2–71.1) mm^2^ larger CSA than the 'low' group.

**Conclusions:**

We suggest that participation (≥ 1/week) in one or more high-impact sports in adulthood is associated with larger vertebral size, and thus increased vertebral strength, among middle-aged women.

## Background

Osteoporosis is a global burden in the ageing population, leading to vertebral fragility [1, 2]. Since vertebral strength is determined not only by its architectural and material properties but also its size [3], knowledge on the modifiable factors that influence vertebral size during life course would be beneficial for preventing vertebral fractures in older age [4].

Physical activity (PA) exposes bone tissue to biomechanical loading and affects bone density, shape and size according to exercise modality [5–7]. Mechanical stress is associated with increased cross-sectional area (CSA) and bone mineral density (BMD) of long bones [8–11]. The extrapolation of these results to vertebrae may be complicated, as bones respond to loading in a site-specific manner [9, 12], and peripheral skeleton and vertebrae have different embryological backgrounds [13]. However, despite the evidence being inconclusive [14], PA has been reported to increase the strength of the lumbar spine [15–17], and an association between physical loading and increased vertebral dimensions has also been reported [18]. In our recent study, we showed that lifelong leisure-time physical activity (LTPA) was associated with larger vertebrae among women, but not among men [19].

High-impact exercise, such as aerobics and running, is characterized by rapid, bone matrix-affecting motions and overall movement [5, 6]. It has been suggested that those who participate in high-impact exercise have increased BMD in most skeletal sites, including the lumbar spine [8, 20, 21], although the association is debatable [10]. There is lack of research on the association between impact exercise and vertebral size instead of BMD.

In this study, we aimed to clarify the association between high-impact exercise and vertebral dimensions. A population-based birth cohort was used to study the participation in high-impact sports at 31 and 46 years of age, and vertebral dimensions in midlife. We hypothesized that participating in high-impact sports was associated with larger vertebrae.

## Methods

### Study population

The Northern Finland Birth Cohort 1966 (NFBC1966) is a prospective population-based birth cohort study [22]. The population initially consisted of pregnant women living in the northern provinces of Finland (Oulu and Lapland) with expected dates of delivery in 1966 (*n* = 12,068 mothers, *n* = 12,231 children, 96% of all births). The mothers and their children have been followed since.

### Progression of the study

**Fig. 1:**
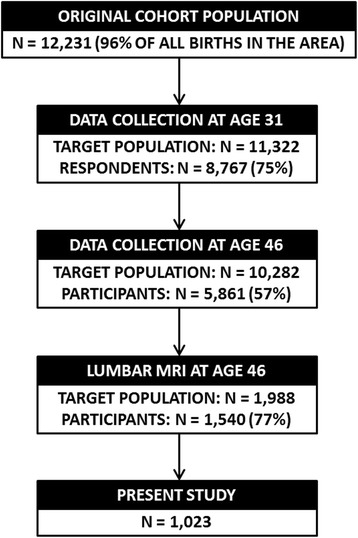
Flow chart of the study population

Figure 1 presents a flow-chart of the study. At age 31, i.e. in 1997–1998, a postal questionnaire enquiring about the participants’ lifestyle and health were sent to all those whose addresses were known. The response rate was 75% (*n* = 8767). At age 46, i.e. in 2012–2014, participants with known Finnish addresses (*n* = 10,282) were again invited to fill in questionnaires and also to take part in clinical examinations, with a total number of 5861 (57%) attendants. Those who attended the clinical examinations and were living in the Oulu region (*n* = 1988) were further invited to lumbar magnetic resonance imaging (MRI). The MRI population consisted of 1540 participants, as 448 participants did not participate in the imaging due to 1) no show (*n* = 409), 2) claustrophobia (*n* = 35), 3) severe obesity (*n* = 3), or 4) a pacemaker (*n* = 1). After the imaging, 517 participants were further excluded from this study due to 1) vertebral pathologies (segmentation error, severe disc degeneration, endplate erosions, presence of spondylodesis or Schmorl’s nodes; *n* = 159), 2) bone-affecting medication (calcium supplements, osteoporosis medication; *n* = 42), and 3) missing exercise or covariate data (*n* = 316). The final eligible population was 1023 participants.

### Assessment of sports participation (exposure)

Sports participation was self-reported at 31 and 46 years of age via identically formulated questionnaires at both time points. The participants were asked about their participation in walking, cycling, cross-country skiing, swimming, running, gym training, aerobics and gymnastics. They were also asked about their participation in ballgames such as badminton, volleyball, tennis and squash (Group #1), and floor ball, ice hockey, soccer, rink ball, and basketball (Group #2). The answer options in each activity were 1) never, 2) once a month, 3) 2–3 times a month, 4) once a week, 5) 2–3 times a week, and 6) ≥ 4 times a week. Those who reported participating ≥1/week were considered ‘participants’ of the respective sport, and others were considered ‘non-participants’.

**Fig. 2:**
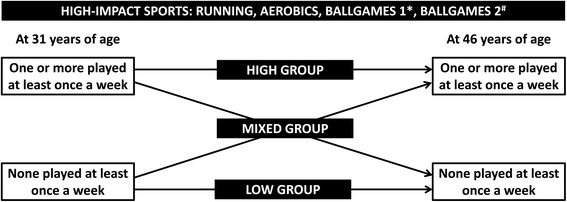
Classification of individuals into ‘high’, ‘mixed’ and ‘low’ impact groups on the basis of their self-reported sports participation at the ages of 31 and 46. *Badminton, volleyball, tennis, squash. ^#^Floor ball, ice hockey, soccer, rink ball, basketball

To estimate high-impact exercise activity, we assigned a longitudinal impact classification to each participant, based on their high-impact sports participation at the ages of 31 and 46 (Fig. 2). For this purpose, a distinction between high-impact and low-impact sports was made a priori; ballgames, running and aerobics were considered high-impact sports [5, 6, 20, 23]. Gymnastics was excluded from the list, as the Finnish term used in the questionnaires was vague. An individual who reported participating ≥1/week in at least one high-impact sport at the respective time point was considered a high-impact sport ‘participant’; the others were considered ‘non-participants’. Individuals who were consistently high-impact sport participants at both 31 and 46 years were placed in the ‘high’ group. Correspondingly, individuals with consistently little activity (< 1/week) in any of the high-impact sports at both time points were placed in the ‘low’ group. The rest of the population, with some high-impact activity, were placed in the ‘mixed’ group.

### Lumbar magnetic resonance imaging and vertebral dimensions (outcome)

We performed magnetic resonance imaging scans using a 1.5-T imaging system (Signa HDxt, General Electric, Milwaukee, WI). The imaging sequences followed routine lumbar spine protocol including T2-weighted fast-recovery fast spin-echo (frFSE) images in sagittal (TR/effTE 3500/112 ms, 4 averages, FOV 280 × 280 mm, acquisition matrix 448 × 224, slice thickness 3 mm with 1 mm interslice gap) and transverse planes (TR/effTE 3600/118 ms, 4 averages, FOV 180 × 180 mm, acquisition matrix 256 × 224, slice thickness 4 mm with 1 mm interslice gap).

**Fig. 3:**
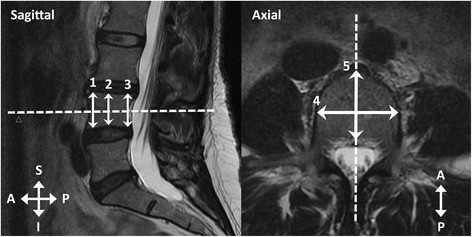
Measured vertebral dimensions. Sagittal view: Anterior height (Measurement 1), posterior height (Measurement 3), minimum height (Measurement 2). Axial view: Minimum mediolateral width (Measurement 4), maximum mediolateral width (not shown); Depth, i.e. anteroposterior length, superiorly (not shown), halfway (Measurement 5) and inferiorly (not shown). Dashed lines indicate corresponding planes. A = anterior, I = inferior, P = posterior, S = superior direction

We measured eight dimensions (in millimetres, mm) of the corpus of the fourth lumbar vertebra (L4) to calculate its axial cross-sectional area and mean height (Fig. 3). L4 was chosen since it is located caudally in the spine but is more stabile than L5 [24]. L4 has been commonly used in studies similar to the present one [19, 25–27]. Height dimensions (anterior height, posterior height, minimum height) were measured using the sagittal view and the midsagittal MR slice. Width dimensions, i.e. minimum mediolateral width and maximum mediolateral width, were measured using the appropriate axial slices, which varied between subjects. Depth dimensions, i.e. anteroposterior dimensions, were also measured using axial slices; the superior depth dimension was measured from the most superior and the inferior depth dimension from the most inferior slice possible. The middle depth dimension was measured from the slice existing halfway.

CSA values were calculated using the acknowledged formula CSA = π * a * b, where a = vertebral width/2 and b = vertebral depth/2 [28]. We used the mean of maximum and minimum mediolateral dimensions as the width dimension, and the mean of superior, inferior and middle anteroposterior dimensions as the depth dimension. Vertebral height was calculated as the mean of anterior, posterior and minimum heights.

The same researcher took all measurements using the NeaView Radiology software version 2.31 (Neagen Oy, Oulu, Finland) prior to data analyses. In order to calculate the intraclass correlation coefficient and measurement errors, 400 MRI measurements were taken for a second time by the original measurer, as described in our previous paper [19].

### Assessment of socioeconomic status, smoking habits and body mass index (covariates)

The number of education years and smoking habits were elicited at 46 years using questionnaires. Socioeconomic status, represented by education years, was classified on a three-point scale (≤ 9 years, 9–12 years, > 12 years). This was determined by asking ‘What is your basic education? 1) Less than nine years of elementary school, 2) elementary school, 3) matriculation examination’. Smoking habits were elicited using two questions: 1) ‘Have you ever smoked cigarettes (yes/no)?’ and 2) ‘Do you currently smoke (yes/no)?’ Three categories were formed on the basis of the answers: 1) non-smoker, 2) former smoker, and 3) current smoker. Body mass index (kg/m^2^) was calculated for each participant using height and weight values that were systematically measured by a trained study nurse at the clinical examinations at age 46.

### Statistical analysis

Statistical analyses were conducted using SPSS software version 22 (IBM, Armonk, NY, USA). *P* values of <0.05 were considered statistically significant. We calculated the descriptive statistics of each variable separately for men and women, and gave them as means and standard deviations (continuous variables with normal distributions) or frequencies and percentages (categorical variables).

As our main approach, we used linear regression analysis to investigate the association between vertebral CSA and impact categories. The CSA of L4, normally distributed among each sex, was the outcome variable, and the impact category variable acted as the predictor in the models. Both crude and adjusted regression analyses were run. Since both CSA [19, 29] and exercise levels [19, 30, 31] were a priori known to differ between sexes, the potential interaction between sex and impact exercise was investigated in the models. The assumptions of linear regression were fulfilled. We gathered beta estimates (β) of the predictors with their 95% confidence interval (CI). The low-impact category was chosen as the reference group.

As a confirmatory analysis, we also used generalized estimating equation (GEE) modelling to investigate the association between vertebral CSA and high-impact exercise. We did not construct any longitudinal impact categories for the GEE models, as the method itself took into account the non-independence of the high-impact exercise data recorded at two time points. In the GEE models, CSA was the outcome variable with the dichotomous high-impact sport participant/non-participant variable (at 31 and 46 years) as the predictor. The participant/non-participant variable was considered a repeated measurement with both 31 and 46-year data in the models. We ran both crude and adjusted analyses, and gathered β estimates with their 95% CIs. The non-participants formed the reference group.

The following variables were assessed as potential covariates in the models: 1) height of L4 (continuous variable) 2) BMI at the age of 46 (continuous variable), 3) education years (categorical variable), and 4) smoking status (categorical variable). We chose these variables for assessment because the literature has suggested they are potential confounders [3, 32–34] and because they were available to us. Each potential confounder was analysed a) individually in relation to vertebral CSA, and b) as part of the linear regression/GEE models. The confounders that were not significantly associated with the outcome and did not alter the models were excluded from the final analyses.

### Ethics

The study adhered to the Declaration of Helsinki and was carried out in accordance with the relevant guidelines and regulations. Approval for the study was obtained from the Ethics Committee of the Northern Ostrobothnia Hospital District. Participation was voluntary and members signed their informed consent at all stages. All data were handled anonymously.

## Results

### Study sample

Our sample consisted of 459 (44.9%) men and 564 (55.1%) women with the mean imaging age of 46.8 (standard deviation 0.4) years (Table 1). Most participants had attended school for 9–12 years and had a BMI ≥ 25. Current or previous regular smoking was reported by 50% of men and 39% of women.

**Table 1:** Characteristics of the sample (*n* = 1023)

		Men (*n* = 459, 44.9%)	Women (*n* = 564, 55.1%)
Age at imaging (years)^a^		46.8 (0.4)	46.8 (0.4)
Cross-sectional area of L4, (cm^2^)^a^		13.3 (1.7)	10.5 (1.3)
Height of L4 (cm)^a^		2.8 (1.5)	2.7 (1.4)
Height (cm)^a^		178.7 (6.1)	164.7 (5.8)
Weight (kg)^a^		86.2 (12.5)	71.8 (14.4)
BMI (kg/m^2^)^a^		27.0 (3.6)	26.5 (5.3)
BMI, classified^b^			
	< 18.5	0.4 (2)	0.7 (4)
	18.5–24.9	32.0 (147)	46.5 (262)
	25.0–29.9	49.2 (226)	31.0 (175)
	≥ 30	18.3 (84)	21.8 (123)
Education (years)^b^			
	≤ 9	2.8 (13)	2.0 (11)
	9–12	73.2 (336)	72.9 (411)
	> 12	24.0 (110)	25.2 (142)
Smoking^b^			
	Non-smoker	49.7 (228)	61.0 (344)
	Former	35.3 (162)	23.8 (134)
	Current	15.0 (69)	15.2 (86)
High-impact sports at age 31^b^			
	Non-participant	56.9 (261)	68.6 (387)
	Participant	43.1 (198)	31.4 (177)
High-impact sports at age 46^b^			
	Non-participant	63.0 (289)	70.7 (399)
	Participant	37.0 (170)	29.3 (165)
Longitudinal impact category^b^			
	Low	44.7 (205)	58.3 (329)
	Mixed	30.5 (140)	22.7 (128)
	High	24.8 (114)	19.0 (107)

^a^Mean (standard deviation)

^b^Per cent (n). BMI = body mass index

### Participation in sports

In our sample, the most frequent types of activity were traditional Finnish outdoor activities such as walking, running, cycling, and cross-country skiing (Table 2). Participation in one or more high-impact sports was reported by 43% of men and % of women at the age of 31, and by 37% of men and 29% of women at the age of 46 (Table 1). A minority of individuals remained active in high-impact sports at both time points (Table 1).

**Table 2:** Study participants’ activity in sports

		At age 31		At age 46	
		Men (n = 459)	Women (n = 564)	Men (*n* = 459)	Women (*n* = 564)
Walking^a^					
	Non-participant	53.8 (247)	29.6 (167)	43.8 (201)	26.2 (148)
	Participant	46.2 (212)	70.4 (397)	56.2 (258)	73.8 (416)
Cycling^a^					
	Non-participant	50.5 (232)	38.7 (218)	65.1 (299)	48.6 (274)
	Participant	49.5 (227)	61.3 (346)	34.9 (160)	51.4 (290)
Cross-country skiing^a^					
	Non-participant	79.1 (363)	84.0 (474)	73.2 (336)	74.3 (419)
	Participant	20.9 (96)	16.0 (90)	26.8 (123)	25.7 (145)
Swimming^a^					
	Non-participant	92.6 (425)	90.4 (510)	93.2 (428)	89.2 (503)
	Participant	7.4 (34)	9.6 (54)	6.8 (31)	10.8 (61)
Running^a,b^					
	Non-participant	77.6 (356)	86.3 (487)	76.7 (352)	82.3 (464)
	Participant	22.4 (103)	13.7 (77)	23.3 (107)	17.7 (100)
Gym training^a^					
	Non-participant	83.2 (382)	90.6 (511)	79.7 (366)	75.9 (428)
	Participant	16.8 (77)	9.4 (53)	20.3 (93)	24.1 (136)
Aerobics^a, b^					
	Non-participant	99.6 (457)	84.4 (476)	99.3 (456)	90.1 (508)
	Participant	0.4 (2)	15.6 (88)	0.7 (3)	9.9 (56)
Gymnastics^a^					
	Non-participant	93.5 (429)	78.9 (445)	92.8 (426)	76.8 (433)
	Participant	6.5 (30)	21.1 (119)	7.2 (33)	23.2 (131)
Badminton/volleyball/tennis/ squash^a, b^					
	Non-participant	83.9 (385)	91.1 (514)	94.3 (433)	94.1 (531)
	Participant	16.1 (74)	8.9 (50)	5.7 (26)	5.9 (33)
Floor ball/ice hockey/soccer/rink ball/basketball^a, b^					
	Non-participant	79.5 (365)	99.1 (559)	85.4 (392)	98.2 (554)
	Participant	20.5 (94)	0.9 (5)	14.6 (67)	1.8 (10)

^a^Per cent (n)

^b^Classified as high-impact

### Vertebral size

The mean CSA of L4 was 13.3 (standard deviation 1.7) cm^2^ among men and 10.5 (1.3) cm^2^ among women. The level of intra-rater reliability was high (intraclass correlation coefficient = 0.963), and the values of relative measurement error (%) were distributed normally around the mean of 0.0, with a standard deviation of 4.9.

### Association between high-impact exercise and vertebral size

A significant sex*impact exercise interaction was detected in the linear regression/GEE models, and all analyses were therefore stratified by sex. Assessment of the potential confounders revealed that vertebral height and BMI were significantly associated with vertebral CSA in the models, and that education years and smoking were not. Education years and smoking did not alter the estimates of the models in any direction and were thus not included in the final models.

The linear regression models showed that the women in the ‘mixed’ and ‘high’ impact groups had larger vertebral CSA than those in the ‘low’ group (Table 3). According to the β estimates of the adjusted models, the mixed group had 36.8 mm^2^ (3.5%) larger CSA and the high group 43.2 mm^2^ (4.1%) larger vertebral CSA than the low group. No statistically significant differences in vertebral CSA were detected among men. In the GEE, participation in high-impact sports was also associated with larger vertebral CSA among women but not among men (Table 4).

**Table 3:** Comparisons between adulthood impact groups (high, mixed, low) in terms of midlife vertebral CSA. Results from linear regression models

	Men (*n* = 459)				Women (*n* = 564)			
	Crude		Adjusted^a^		Crude		Adjusted^a^	
	β	(95% CI)	β	(95% CI)	β	(95% CI)	β	(95% CI)
Adulthood impact group								
Mixed vs. low	−2.1	(−39.8; 35.6)	2.1	(−33.8; 38.1)	19.5	(3.3; 59.2)	36.8	(11.2; 62.5)
High vs. low	−5.6	(−45.7; 34.6)	5.0	(−33,7; 43.7)	27.6	(1.5; 53.8)	43.2	(15.2; 71.1)

^a^Adjusted for vertebral height and BMI at age 46. *β* Beta estimate (mm^2^). *CI* confidence interval

**Table 4:** Results from GEE models analysing the association between longitudinal impact exercise (31 to 46 years) and midlife vertebral CSA

	Men (*n* = 459)				Women (*n* = 564)			
	Crude		Adjusted^a^		Crude		Adjusted^a^	
	β	(95% CI)	β	(95% CI)	β	(95% CI)	β	(95% CI)
Participation in impact exercise								
Participant vs. non-participant	−3.7	(−30.7; 23.3)	3.3	(−21.9; 28.6)	25.4	(5.3; 45.6)	34.3	(14.6; 54.0)

^a^Adjusted for vertebral height and BMI at age 46. *β* Beta estimate (mm^2^). *CI* Confidence interval

## Discussion

The aim of this cohort study was to reveal the association between high-impact exercise in adulthood and vertebral size in midlife. According to our results, high-impact exercise was indeed associated with large vertebrae, but only among women. Men had similar vertebral dimensions regardless of whether or not they participated in high-impact sports.

Our findings regarding the CSA differences between impact categories were rather moderate (3—4%). However, a systematic review of case-control studies has shown that the intact vertebrae of individuals with vertebral fractures are 1.2—14.2% smaller in CSA than those of individuals with no vertebral fractures [3]. Falling well within this range, our results thus seem clinically relevant. It must be acknowledged, however, that the detected association between high-impact exercise and vertebral size may have been stronger at an earlier age. Unfortunately, we had no data regarding this population’s sports participation before the age of 31. Vertebral growth is known to continue until the peak bone mass will be reached, i.e. well beyond adolescence [35], which potentially explains why the beneficial association was detected in adulthood.

In this study, we observed that participation in high-impact sports was associated with vertebral size in a sex-related manner. This finding is in line with our previous study conducted on the same middle-aged cohort population with the conclusion that LTPA has a small positive effect on vertebral size among women only [19]. As discussed in the previous paper, vertebral size (both absolute and relative to body size) is smaller among women, which might suggest that there is a higher ability or a greater demand for the female vertebra to enlarge in size. Sex-related differences in the rate of periosteal apposition [3, 36] may explain the observed findings. Men seemed to be more active in high-impact sports, indicating that sex-related differences in exercise habits may also be reflected in our results.

We excluded gymnastics from the list of high-impact sports due to the vagueness of the term that was used in the questionnaires. The Finnish word ‘voimistelu’ can be interpreted as denoting either professional gymnastics, or low-intensity calisthenic exercises performed e.g. at home. Although there were rather few gymnastics participants in our sample, we acknowledge that this exclusion is a limitation of the study. Gymnastics should be considered a high-impact sport in future studies, and its association with vertebral size should be investigated.

As members of a 1966 birth cohort, the subjects were born in the same year (minimizing the confounding effect of age), and had reached their skeletal maturity so that the possible long-term effects of exercise on vertebral bone were more likely to be prevalent. Our sample size was rather large compared to previous research on vertebral dimensions [3]. Although this study focused on vertebral size, we acknowledge that exercise may affect vertebral mineral and architectural properties, thus contributing to its strength. However, our previous study found no association between LTPA and trabecular bone density parameters in vertebrae [27].

L4 was chosen as our vertebra of interest, as it typically is more stable than L5 [24]. Although we have previously demonstrated the high accuracy of MRI in measuring vertebral size [37], the location of L4 varies among individuals and may have affected our slice orientation, adding to the measurement error. Our earlier comparisons [19] and other literature [38] suggest that our study is comparable with those that have utilized other lumbar segments.

## Conclusions

We conclude that participation in high-impact sports during adulthood is associated with large midlife vertebrae and thus also increased vertebral strength among women. However, the detected differences were of minor magnitude, and high-impact sports were not associated with vertebral size among men. More extensive research is needed to confirm these findings and to explain the observed sex-related differences.

## Abbreviations

BMD: Bone mineral density
BMI: Body mass index
CI: Confidence interval
CSA: Cross-sectional area
effTE: Effective echo time
FOV: Field-of-view
frFSE: Fast-recovery fast spin-echo
GEE: Generalized estimating equation
IQR: Interquartile range
L4: Fourth lumbar vertebra
LTPA: Leisure-time physical activity
MRI: Magnetic resonance imaging
NFBC1966: Northern Finland Birth Cohort 1966
PA: Physical activity
SD: Standard deviation
Β: Beta estimate
